# Versatile open-source fluorescence documentation system

**DOI:** 10.1016/j.ohx.2023.e00450

**Published:** 2023-06-29

**Authors:** Leonhard Bandilla

**Affiliations:** Pfahlweg 18A, 82346 Andechs, Germany

**Keywords:** Fluorescence imaging, Gel electrophoresis, Open hardware, Macroscopic imaging

## Abstract

Fluorescence has long been the best method for detecting bio-molecules at high sensitivity and with the possibility of easy data analysis and is routine for gel electrophoresis and much more. However, the systems for detecting the fluorophores remain expensive and thus inaccessible to many. Most commercial systems are often only optimized for one specific application, making reuse difficult. This paper demonstrates, characterizes and evaluates an inexpensive and versatile system for the detection of fluorophores with two wavelengths using high power LEDs. The LEDs are arranged in two banks angled downward at a tray in a way that allows for bright and uniform illumination while preventing direct reflections into the camera. The emitted light is filtered through an exchangeable filter frame and can be detected using the camera of a smartphone or similar device. By using filters both in front of the LEDs and the camera, very low background and using sufficient exposure times, very high sensitivity can be achieved. The two wavelengths of excitation light and the exchangeable filters allow for optimization for the specific fluorophore used and thus highest brightness.

## Graphical abstract

**Figure:**
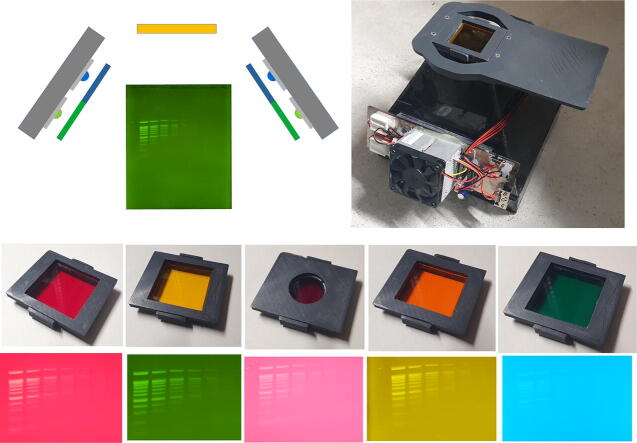

**Table:** Specifications table

Hardware name	Versatile Open-source Fluorescence Documentation System
Subject area	- Biological sciences (e.g., microbiology and biochemistry)
Hardware type	- Imaging tools
Closest commercial analog	Commercial gel documentation systems
Open source license	CC-By Attribution 4.0 International
Cost of hardware	100–200€ depending on the configuration
Source file repository	https://doi.org/10.17605/OSF.IO/FCQNR

## Hardware in context

When fluorescence was first used to detect DNA for gel electrophoresis, the most common stain was ethidium bromide. This fluorescent stain was often excited using UV light, mostly by using UV tubes arranged underneath the gel in a so called transilluminator. Due to safety concerns regarding the possible mutagenicity of ethidium bromide, newer stains were developed, these often have different spectra and make excitation possible using visible light [1], [2], [3]. Together with the development of affordable high power LEDs, this lead to many designs adapting green or blue LEDs illuminating the gel either from underneath or from the sides [4]. These devices are often more sensitive, safer and avoid UV damage to the DNA. However, the price for many of the commercial devices remains high.

It is a common project to build a simple gel documentation system using blue LEDs and colored acrylic as filters [5], [6]. However, many of those devices suffer from high and uneven background as well as low sensitivity due to excitation light reaching the sensor. Their simplicity, ease of construction and cost however are unmatched and the sensitivity is often sufficient for application like PCR that produce strong bands. The reason for this can be direct reflections or even a direct line of sight to the LEDs or inappropriate filtering of the excitation light [7]. LEDs often have a broad spectrum and without filters, this often overlaps the very dim fluorescence and gets captured as background. Other designs use commercial transilluminators and have achieved relatively high sensitivity at a low cost [8], [9], [10]. However this assumes that a transilluminator is already available. Other approaches using high power LEDs have been done, however often the construction wasn’t light tight [11]. In many cases filtering of the excitation light however is neglected which reduces cost but results in high background. Also many design power the LEDs by simple series resistors which is very cost effective but can lead to intensity fluctuation during time lapse experiments, compared to a constant current driver that is more stable.

For this reason, even for LED illumination, filter cubes that incorporate both excitation as well as emission filters and a dichroic beam splitter have been the standard for fluorescence microscopy. A recent approach to use filter cubes for macro imaging applications has allowed for multi-channel imaging of fluorescent bacteria [12]. While filter cubes lead to very high sensitivity, low background and multi-channel imaging, they require at least one dichroic beam splitter, which usually is more expensive than glass or even colored plastic filters.

**Fig. 1:**
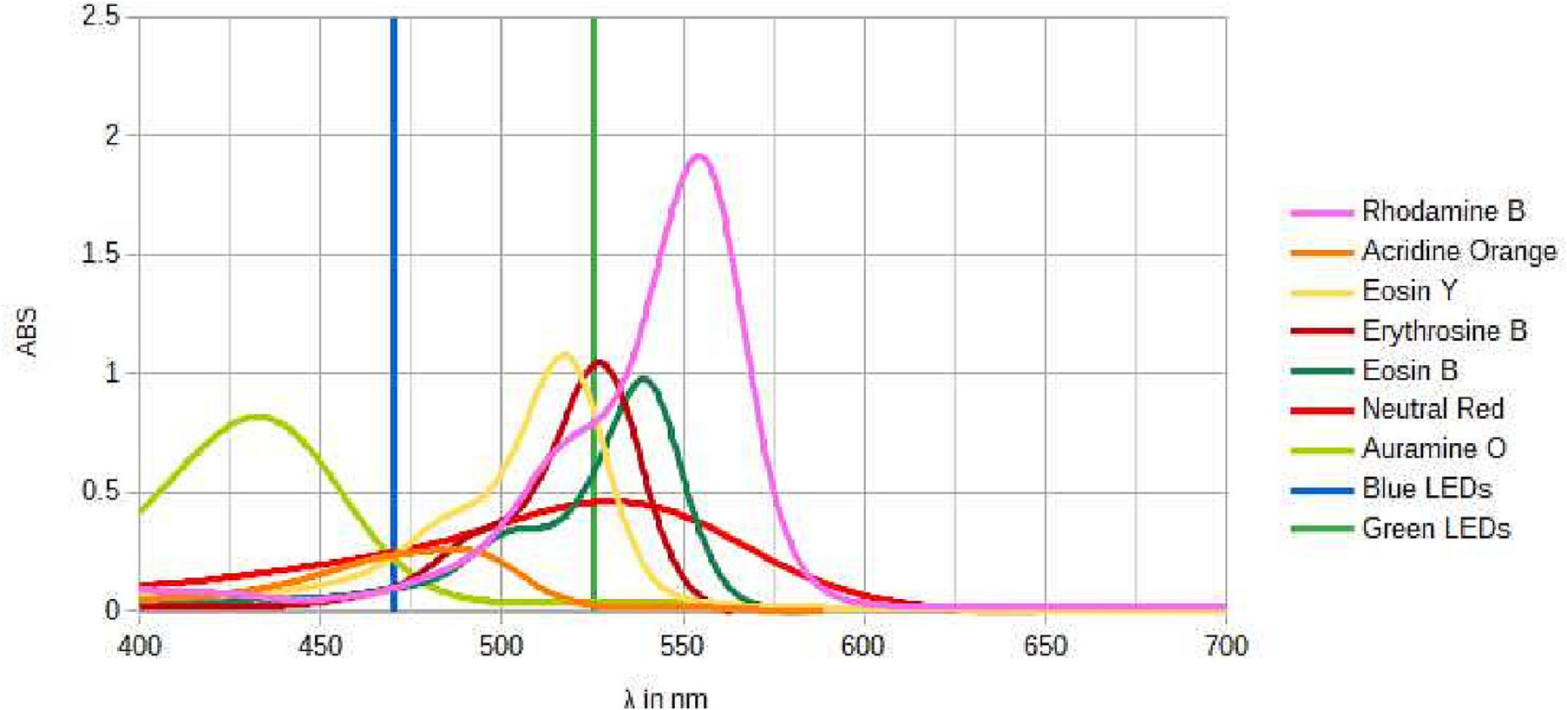
Absorption spectra of the fluorescent dyes tested at a concentration of 10 µg/ml, apart from GelRed, as well as the wavelengths of LEDs used, note that the absorption is logarithmic and not normalized.

The ideal excitation wavelength for many of the modern gel stains as well as Acridine Orange is blue light with 470 nm LEDs matching the absorption spectrum well [13]. However, many gel stains with a spectrum similar to ethidium bromide can also be excited using green light with 525 nm LEDs being a good option. As can bee seen by Fig. 1, many other fluorophores can also be excited using this wavelength, combining both excitation wavelengths which allows for increased versatility and has been done commercially [14].

## Hardware description

**Fig. 2:**
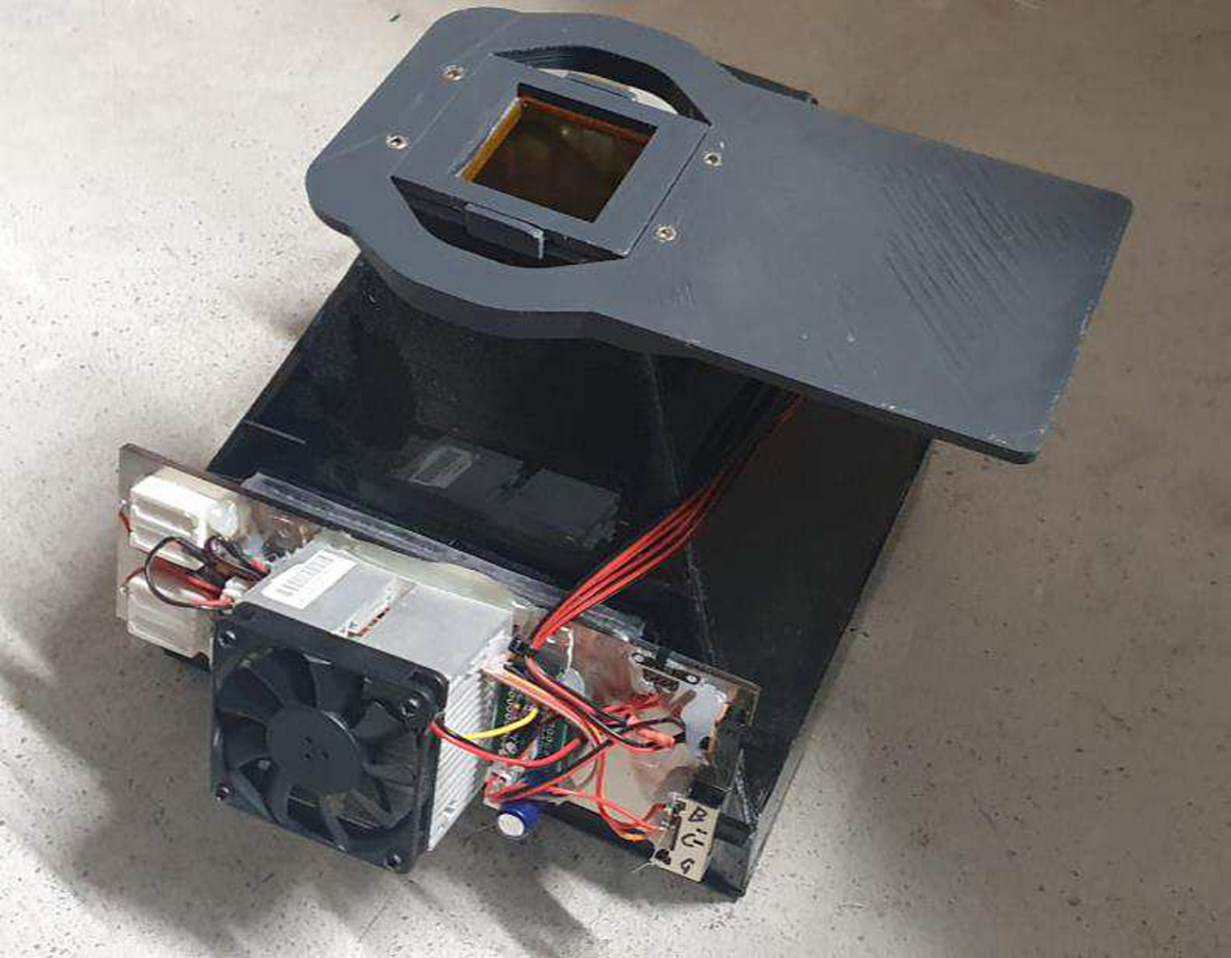
Fully assembled imager with the OG530 filter mounted as well as the smartphone platform.

The imager presented here, like many fluorescence imaging systems, can be simplified to two excitation light sources with their excitation filters and a set of emission filters that block the excitation light. The excitation light source are high power LEDs that are mounted in two banks on the broad sides of the imager. LEDs with two wavelengths are used that are arranged in a 2 × 4 checkerboard pattern with excitation filters mounted directly in front of the LEDs in the same pattern. The emission filters are mounted each in a frame that uses a simple snap mechanism to exchange the filters. Unlike other systems, this imager does not need a beam splitter for the excitation light. The two banks of LEDs are angled downwards at the sample at an angle that prevents direct reflections into the camera which could cause uneven background. The sample is placed on a removable tray that is loaded into the device from the front. The base of the device is a cuboid that houses the sample tray with a pyramid on top that has the LED banks placed on the sides with the emission filters. The camera is placed on top. While most of the device is made out of 4 mm polycarbonate, the top piece that hold the filters, the filter frames themselves and the optional platform that holds the camera are 3D-printed from ABS plastic. With the use of this platform, a smartphone can be stably placed on top for long exposures. The inside of the device and the tray are painted black and the partly covered by black tape to increase the opacity and shield from outside light. Fig. 2 shows the device fully assembled with a filter and camera platform installed, both printed in gray ABS.

The electronics consist of simple linear regulators for the LEDs with shunt resistors so that a constant current of 1.4A with two banks of 4 series LEDs in parallel is kept. Switching regulators were avoided due to the higher cost and complexity, it was later also determined that this would only improve the efficiency by about 13% in a worst case scenario. The banks of LEDs with different wavelengths are driven separately due to the need for independent control and the different forward voltages. Due to the large amount of waste heat produced, the LEDs, all linear regulators as well as their shunt resistors are mounted with thermal glue to a 4 mm thick steel plate with a cooler to dissipate the heat. The fans of the coolers are supplied with 12 V using another linear regulator. This is atypical for most DIY imagers as usually less powerful LEDs are used. However, the very bright LEDs minimize the influence of stray light entering the device and reduce exposure times and allow for visibility of most signals with the bare eye. A thermal test proved that the cooler is necessary to keep the LEDs at safe temperatures. The device uses switches to select the wavelength and to switch on the device. The fans can also be switched off using another switch, this should be limited to short periods of time as shown in the thermal test.

A big difference between this device and most commercial gel imagers is that it illuminates the sample from the top while most gel imagers illuminate from the bottom through a glass plate. While this option was also evaluated, it comes with some issues. The device would have to be much thicker for an even distribution of the light. It would also result in an easy to scratch plastic sheet or a fragile and expensive glass sheet. The biggest downside though would be that such a setup is exclusively usable with gels, limiting the usability for other purposes. The device presented here can not only image gels but also TLC plates with fluorescent compounds, microtiter plates and generally any object that fits into the tray and has some fluorescent property. The disadvantage is that if gel extractions are to be performed, it can only be done by measuring the position of the band and cutting the gel outside of the imager, unlike a transilluminator that allows for cutting of the gel while visualizing the band to be cut.

**Fig. 3:**
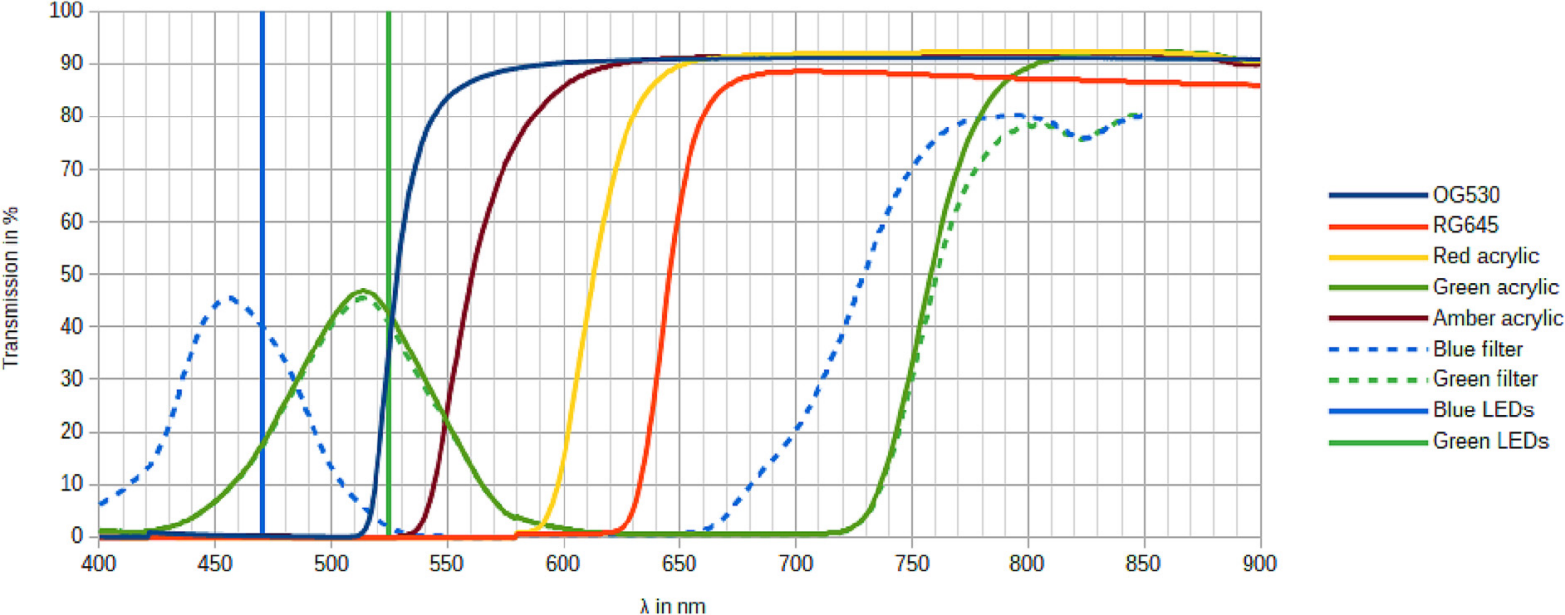
Overview of the spectra of the filters used, the emission filters are shown with solid lines and the excitation filters with dashed lines. The center wavelnghths of the LEDs are shown as vertical lines, although one should be aware that this is only the center wavelength and that LEDs aren't monochromatic.

Compared to many other DIY imagers the biggest difference is the use of very high powered LEDs. Most other designs use blue low power LEDs, which combined with the strongly fluorescent green gel stains available, usually give a strong enough signal and don’t required cooling. However, as this device is also used to image fluorophores with sub-optimal spectra for the LEDs, like imaging GelRed, a higher intensity is advantageous and necessary. When using filters with cutoffs far away from the emission wavelength of the LEDs a very low background can be achieved, but it also results in a very weak signal. During some tests exposure times up to 30 s were required even at this high power, using lower power LEDs would therefore require unpractical exposure times. Another important design decision was the use of excitation filters for the LEDs. Unlike lasers, the light from LEDs isn’t monochromatic so without excitation filters there could be spectral overlap into the spectrum of the emission filter which can lead to a high background signal. This relationships between the excitation filters and emission filters can be seen in Fig. 3. While this might be acceptable for the strong signal of many green fluorescent gel stains, for some stains and applications higher sensitivity is required.

In general, many of the design decisions like the use of higher power LEDs, good emission filters or the use of excitation filter increased the cost of the device above that many other solutions. However, in the context of operating a normal laboratory and compared to commercial solution that this device can compete with the cost is neglectable. The exchangeable filters increase the versatility and the strong LEDs allow for high sensitivity with strong signals and thus easy visibility, justifying the cost for most applications.

The added weight and power requirements however, drastically limit the usability for field work, however this usually doesn’t pose an issue. The complexity however drastically increases the assembly time and of course cost, however as stated above this decision was made to increase the sensitivity. Due to the placement of the LEDs and the resulting light pattern, this device can not be used for accurate quantification unless the object is very small or compensation is performed. While the filters are easily exchangeable, the LEDs are not. This limits the applicability of the device for other applications that need different excitation wavelengths for example for NIR fluorophores. It would thus be an interesting upgrade to develop a system with exchangeable illumination and possibly use monochromatic light sources to further reduce background and increase sensitivity. This device however balances cost and complexity as well as sensitivity, uniformity and a low background.

Due to its versatility the device can be used for a variety of tasks:

The main use case is agarose gel visualization. With the very bright illumination even sub-optimal gel stains can be used and even very faint bands can be seen. It can replace expensive gel documentation systems or dangerous UV transilluminators in that regard.
Due to the illumination from above, the sample doesn’t have to be transparent. This allows for imaging of TLC plates with fluorescent compounds, charcoal agar plates or any other sample that would usually require a separate device to image.
By using two different wavelengths of LEDs a large range of fluorophores can be utilized without needing a second imager. This increases the flexibility and doesn’t limit reuse for future applications.
Because of the exchangeable filters, both inexpensive colored plastic as well as high quality glass filters can be used. Not only does this increase flexibility and is required when using two excitation wavelengths, but it also lowers the barrier of entry with upgrades being possible in the future.

### Safety considerations

When assembling and testing the LED arrays, never look directly at the LEDs when operating at high power. With careful control of the voltage, the LEDs can be tested at low power. While most of the very bright light is effectively shielded to the outside, some can exit through gaps in the plastic. Avoid looking at bright objects without a filter installed.

The fans can cause injury to fingers, the use of fan guards is recommended for that reason. Avoid loose items of clothing, long hair or loose items like paper towels around the fans. Depending on the heat sink used, it might have sharp edges. While the overall temperature of the steel plates stays below 50 °C under normal conditions, some components, specifically the linear regulators and the large shunt resistors can get dangerously hot.

Many of the fluorophores used during these tests and as typical gel stains intercalate DNA and often are suspected mutagens and carcinogens. Always follow standard laboratory practice around dangerous chemicals. Specifically be very careful not to contaminate the camera by changing or taking off gloves after handling contaminated liquids and surfaces.

Don’t turn off the fans for extended periods of time as shown in the thermal tests. For the same reason don’t obstruct the fans or the airflow. The power supply can get hot during operation and should not be surrounded by flammable or insulating material.

*Design files*

## Design files summary

**Table:** 

Design file name	File type	Open source license	Location of the file
Electronics.sch	Electronics schematics	CC BY 4.0	Repository
schematics.pdf	Electronics schematics	CC BY 4.0	Repository
GelImager.blend	Blender CAD file	CC BY 4.0	Repository
1InchFilterFrame.stl	STL 3D file	CC BY 4.0	Repository
36x40FilterFrame.stl	STL 3D file	CC BY 4.0	Repository
40x40FilterFrame.stl	STL 3D file	CC BY 4.0	Repository
PhoneHolder.stl	STL 3D file	CC BY 4.0	Repository
TopPiece.stl	STL 3D file	CC BY 4.0	Repository

• Electronics.sch: This design file contains the schematics, including comments on cooling and grouping into PCBs.
• schematics.pdf: a PDF version of the schematics
• GelImager.blend: This design file contains a complete 3D model of the imager and should be used to transfer the dimensions to the polycarbonate sheet.
• 1InchFilterFrame.stl: An STL file of the filter holder for a one inch diameter filter such as the RG645.
• 36 × 40FilterFrame.stl: An STL file of the filter holder for a filter with the dimensions 36 × 40 mm, such as the amber plastic filter used.
• 40 × 40FilterFrame.stl: An STL file of the filter holder for a filter with the dimensions 40 × 40 mm, such as the green and red plastic filter used as well as the OG530.
• PhoneHolder.stl: An STL file of the platform that can be used to set down a camera or smartphone.
• TopPiece.stl: An STL file of the top piece that the filters and the platform attach to

*Bill of materials*

## Bill of materials summary

**Table:** 

Designator	Component	Number	Cost per unit-currency	Total cost -currency	Source of materials	Material type
LED blue	LED 3 W 470 nm	8	0.79€	7.90€ for 10pcs	Ebay	Semi-conductor
LED green	LED 3 W 525 nm	8	0.85€	8.49€ for 10pcs	Ebay	Semi-conductor
Polycarbonate 4 mm	Polycarbobate sheet 4 mm, total 2322.46 cm^2^	1	22.50€	22.50€ for 50 × 60 cm	Ebay	Polymer
Steel plate	Stainless steel plate 59 × 192 × 4 mm	2	12.34€	24.68€	Feld-Eitdorf	Metal
Epoxy glue	Two-part epoxy adhesive	1	13.50€	13.50€	Ebay	Adhesive
Red acrylic	Red tinten acrylic 40 × 40 × 3 mm	1				Polymer
Green acrylic	Green tinten acrylic 150 × 150 × 3 mm	1	2.68€	2.68€	Ebay	Polymer
Blue acrylic	Blue tinten acrylic 150 × 150 × 3 mm	1	2.68€	2.68€	Ebay	Polymer
OG530	Schott OG530 40 × 40 × 2 mm	1				Optical Part
RG645	Schott RG-645 25.4 × 3 mm	1	32.50€	32.50€	Edmund Optics	Optical Part
Orange acrylic	Orange acrylic 36 × 40 × 3 mm	1				Polymer
ABS filament	ABS filament 1.75 mm 100 g	1	5.20€	12.99€ for 250 g	3DJake	Polymer
Glue	Rubber-like glue ∼5 g	1	1.66€	5.99€ for 18 g	Ebay	Adhesive
LM317	LM317t voltage regulator	3	0.95€	2.84€ for 3pcs	Ebay	Semi-conductor
Barrel jack	5.5 mm barrel jack	1	1.19€	1.19€	Ebay	Electrical Component
DC power supply	19 V 4.74A DC power supply	1	15.99€	15.99€	Ebay	Electrical Part
10 Ω Resistor	10 Ω Resistor 5 W	2	0.38€	1.89€ for 5pcs	Ebay	Electrical Component
1 Ω Resistor	1 Ω Resistor 5 W	2	0.38€	1.89€ for 5pcs	Ebay	Electrical Component
Switch SPDT	Slide switch SPDT	2	0.50€	4.99€ for 10pcs	Ebay	Electromechanical Component
Switch DP3T	Slide switch DP3T	1	1.05€	10.49€ for 10pcs	Ebay	Electromechanical Component
100 nF capacitor	100 nF ceramic capacitor	3	cents	cents	Mouser	Electrical Component
1 μF capacitor	1 μF electrolytic capacitor	3	cents	cents	Mouser	Electrical Component
100 μF capacitor	100 μF electrolytic capacitor	1	cents	cents	Mouser	Electrical Component
JST 3 pin male	JST XH 3 pin connector male	1	cents	cents	Ebay	Electromechanical Component
JST 2 pin male	JST XH 3 pin connector male	5	cents	cents	Ebay	Electromechanical Component
JST 3 pin female	JST XH 3 pin connector female	1	cents	cents	Ebay	Electromechanical Component
JST 2 pin female	JST XH 3 pin connector female	5	cents	cents	Ebay	Electromechanical Component
Resistor 2.2 kΩ	1/4W 2.2 kΩ resistor	1	cents	cents	Mouser	Electrical Component
Resistor 240 Ω	1/4W 240 Ω resistor	1	cents	cents	Mouser	Electrical Component
Perfboard	8 × 2 cm Perfboard	1	cents	cents	Ebay	Electrical Component
Twin wire	Twin wire ∼30 cm	7	cents	cents	Ebay	Electrical Component
Thermal glue	Thermally conductive adhesive	1	∼1€	5.89€ for 10 g	Ebay	Adhesive

The minimum width of the steel plate is now 60 mm, this should be acceptable as well, in case it this is issue it has to be sanded to the right dimensions.

The OG530 has been bought second hand and no exact equivalent could be found, a close replacement would be a OG530 with a diameter of 25.4 mm, available from Edmund, the only square filter available would be 50 × 50 mm, which would not fit.

The orange and red acrylic were not specifically bought for this project and no supplier or part number could be found. If possible it is recommended to characterize the spectrum of any plastic to be tested and to compare it to the spectra of the filter used here, raw spectral data of the filters can be found in the repository.

## Build instructions

For building the device as described below, the following additional tools and items are required:

• A jigsaw or scroll saw to cut or the polycarbonate and any plastic filters
• Measuring equipment and a fine waterproof marker
• Coarse sandpaper
• A syringe with a blunt needle and a small amount of dichloromethane or similar solvent to weld the polycarbonate
• Alternative adhesives to the ones listed can be used if evaluated carefully
• Double-sided adhesive tape that can resist shear forces, thick mounting tape works well for this
• Black spray paint
• A voltage and current controlled power supply to test the LEDs
• Soldering equipment
• A crimping tool for the connectors used, alternatively other connectors can be substituted
• A drill press and drill bits suitable for stainless steel
• Hot glue gun and glue sticks
• Wire cutters

### Polycarbonate parts

Using the blender file found in the repository, transfer the measurements to the 4 mm polycarbonate plate. The Blender plugin “MeasureIt” can be used to get precise dimensions [15]. Cut out the polycarbonate as precise and straight as possible, if in doubt, cut the pieces slightly larger and sand them to the correct dimensions using coarse sandpaper. In addition to the two wavelength design, a single wavelength can also be used, the alternative side pieces can be found in the supplied blender file.

**Fig. 4:**
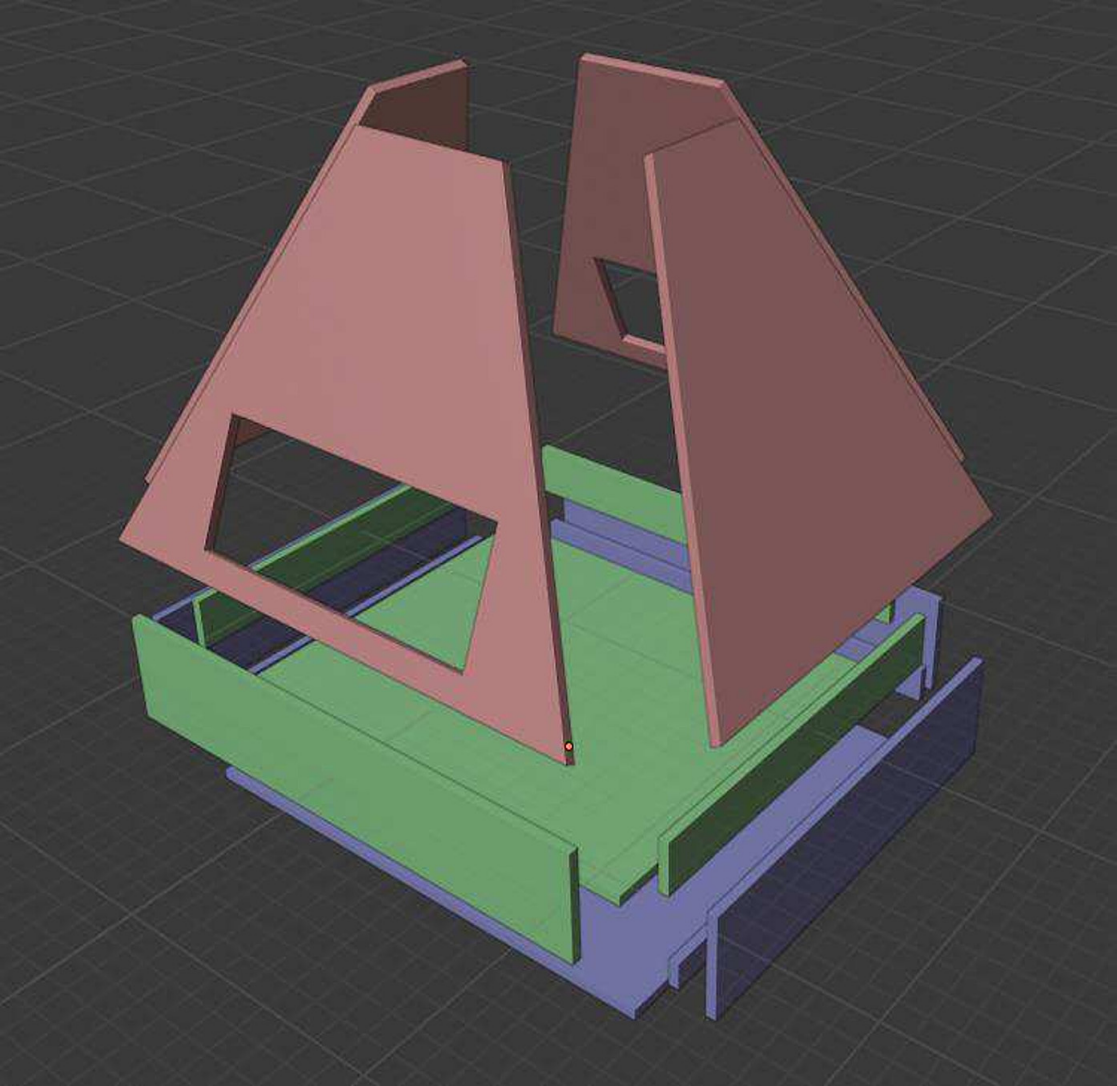
Exploded view of the polycarbonate parts, the top part in red, the lower half in blue and the tray in green. (For interpretation of the references to color in this figure legend, the reader is referred to the web version of this article.)

The parts to be assembled can be seen in Fig. 4. Start assembling the lower half (blue) of the enclosure. Use the blender file to see which surfaces are in contact. Fill a small syringe with dichloromethane with a blunt needle attached. Other solvents can also be used. Warning: Dichloromethane is a possible human carcinogen and acutely toxic as well as a greenhouse gas and ozone depleting. It is very volatile and can easily create a hazardous atmosphere. Perform this part only in a very well ventilated area or fume hood, avoid skin contact and be aware of the glove compatibility when wearing gloves. Use as little dichloromethane as possible and always close bottles tightly. The use of a blunt needle is very important, there have been severe injuries reported from needle stick injuries involving dichloromethane [16]. Add a small amount of dichloromethane to both surfaces to be bonded and wait a short amount of time before adding a bit more. Then firmly press both surfaces together, sliding them back and forth to create better contact. Avoid getting dichloromethane on surfaces not to be bonded as it leads to marks on the plastic. The tray (green) can be assembled similarly.

For the pyramid shaped top part (red), hold the pieces together with tape in the right shape. Use two component epoxy glue to secure the parts, dichloromethane can not be used as the odd angles result in only a very small surface area being in contact with each other. Allow for the glue to harden completely. Never touch the uncured epoxy glue with your bare skin as it is known to be a sensitizer, it is recommended to wear gloves.

At this point spray paint both the top (red) and lower half (blue) was well as the tray (green). In case the paint isn’t opaque enough, black, non reflective tape can be used in addition. Allow for the paint to dry before proceeding.

Using epoxy glue, attach the pyramid (red) to the lower half (blue), making sure the tray (green) can slide in and out freely. Allow for the epoxy glue to harden completely before proceeding, usually waiting overnight is sufficient.

### LED panels

**Fig. 5:**
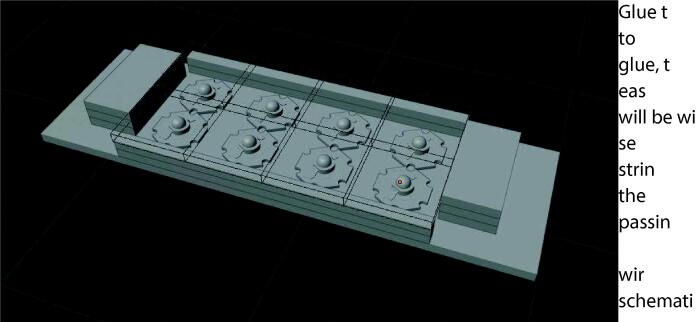
Rendering of the assembled LED panel with the filters shown as wire frames for better visibility.

**Fig. 6:**
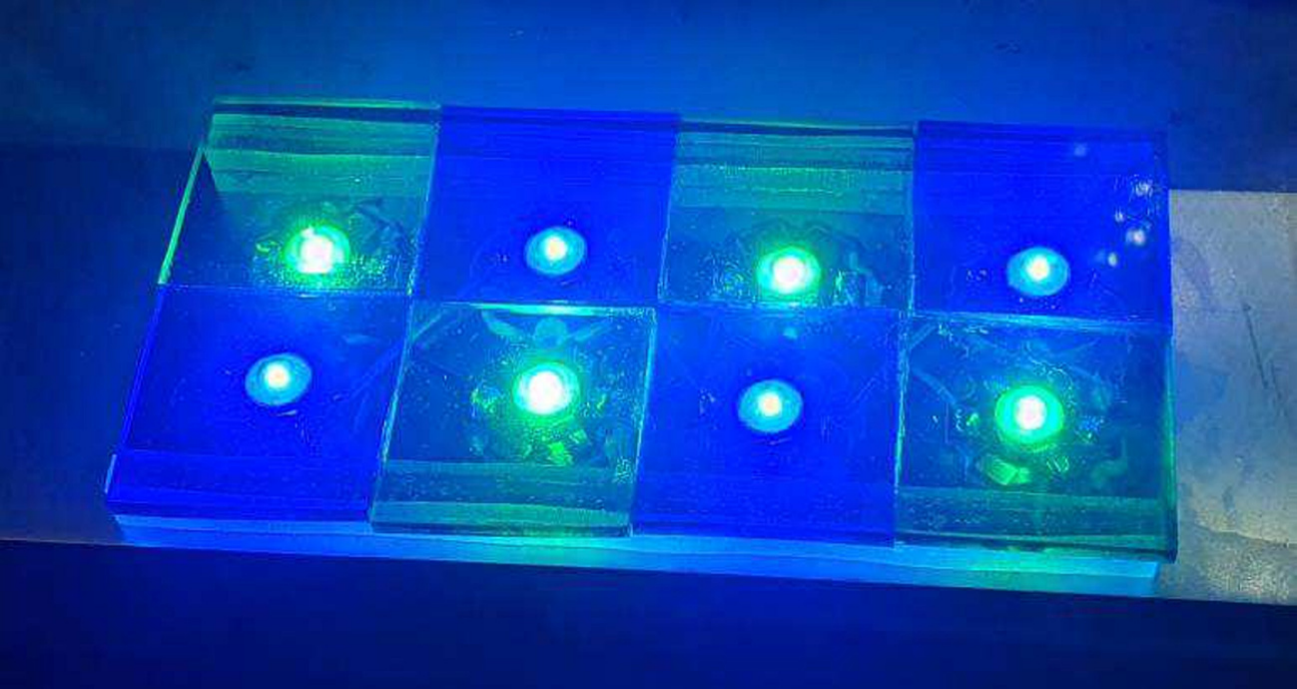
LEDs arranged in a checkerboard pattern with the filters already installed.

From the blender file the positions of the LEDs can be transferred to the metal plate. If using two wavelengths of LEDs, the LEDs should be touching in the middle. Arrange the LEDs in a checkerboard pattern, alternating green and blue LEDs so that when the two panels are mounted, the pattern are mirrored to each other, this pattern can be seen in Fig. 5, Fig. 6. Before gluing the LEDs to the metal plate, drill two holes near the ends of the LED arrays. The size of the hole depends on the wires used, with the wires used in this build, a 3 mm hole was chosen.

Glue the LEDs in the right pattern to the metal plate using thermal glue, taking note of the polarity for easier wiring. Each color of LEDs will be wired with 4 LEDs in series. Solder the LEDs into strings of 4 for each color and use the twin wire on the last LEDs, passing the wire through the holes drilled before. The exact wiring can be seen in the schematics available in the repository. Be careful when soldering anything, the hot tip of the soldering iron as well as its shaft can cause serious burns. Leaded solder is toxic, there is no safe dose of lead and it can cause serious chronic health risks, always wash your hands after handling leaded solder, the author recommends to completely avoid leaded solder. Additionally the fumes produced by the flux used should not be breathed in, the use of a fume extractor is recommended.

Use leftover polycarbonate to make 8 pieces with a length of 12 cm and a width of roughly 0.5 cm. Also make 12 pieces with dimensions of about 4x2cm. The exact dimensions aren’t important, however, too small of a surface area will result in poor adhesion when later attaching the banks of LEDs. Then cut the emission filters out of the panel of colored plastic. Eight squares of 30 mm of both colors are required in total. Arrange the squares matching the checkerboard pattern of the LEDs. Use epoxy to glue the squares into one large filter, dichloromethane cannot be used. Use dichloromethane to glue stacks of 3 of the 4x2cm plates to each other. When the assembled emission filter has hardened, use dichloromethane to attach two of the long pieces stacked on top of each other to each edge of the filter. Then use epoxy glue to attach the filter assembly to the LED bank. Attach the stacks of 2 × 4 cm plates to the outside of the LED arrays using epoxy glue. This assembly can be seen in Fig. 6 with the filters only being shown as outlines and in Fig. 5 fully working. Using strong double sided tape, like mounting tape, the panels can then be attached to the device. Place the panels centered onto the pyramid, if 4 × 2 cm blocks were used, then the blocks upper outside corner should roughly align with the edge of the pyramid.

### Electronics and wiring

**Fig. 7:**
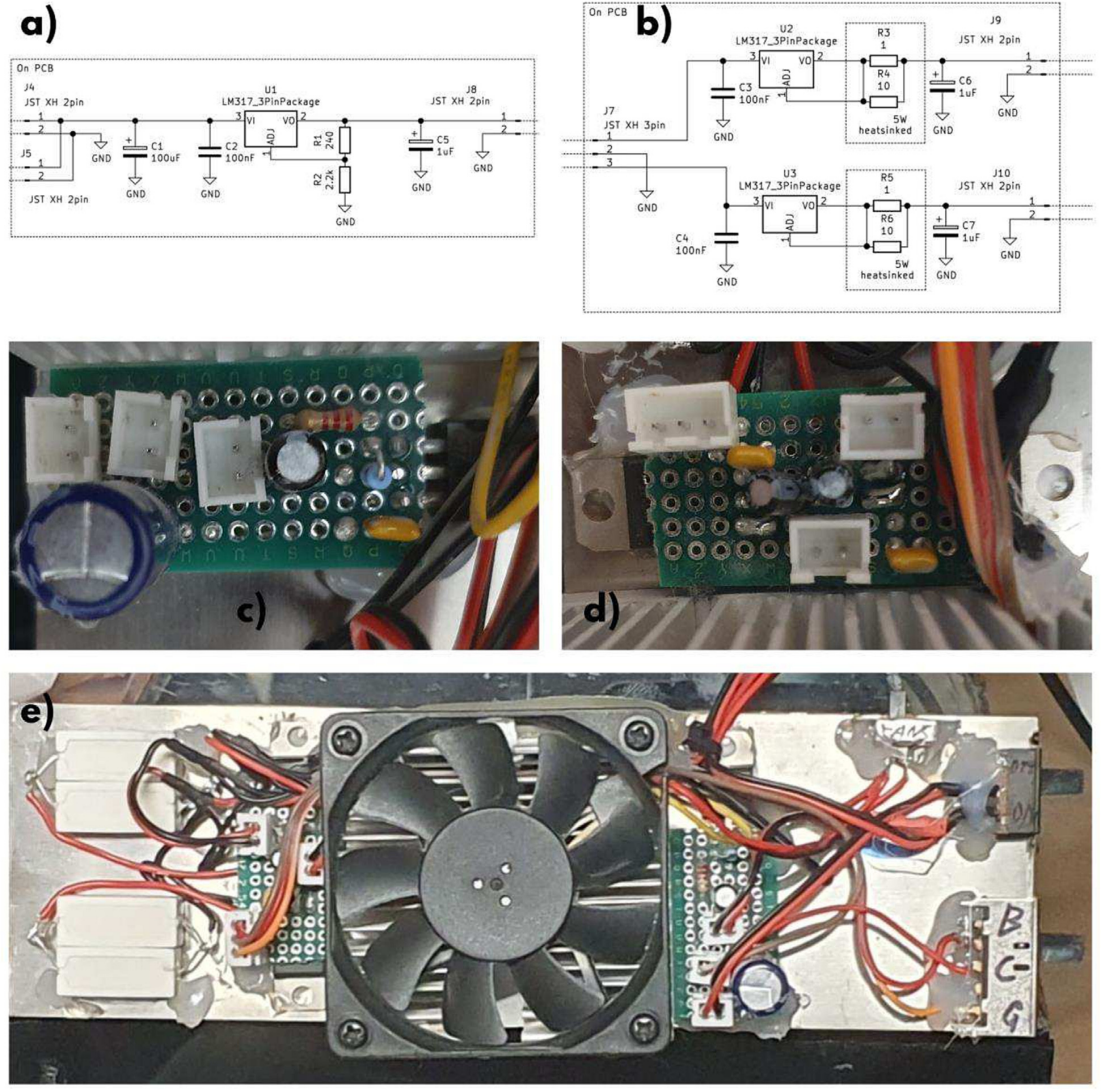
The distribution PCB (a and c) and the LED driver PCB (b and d) with their schematics as well as a general overview of the electronics and wiring (e).

For the schematics the “Electronics.sch” file should be opened using a suitable software such as KiCAD. The PCB layouts shown should only be regarded as a suggestion, depending on the capacitors used, the layout might have to be done differently. Excerpts of the schematic and examples of the PCB layout can be seen in Fig. 7.

Using a pair of wire cutters, cut the perfboard into two halves and trim off the end pieces. Build each of the circuits on their respective PCB, the exact layout doesn’t matter. It is important to mount the voltage regulators on the backside to transfer the heat to the metal plate and to add the shunt resistors using short pieces of wires for the same reason, as seen in e), the shunt resistors can be made into pairs of the1Ω and10Ω resistor by tightly twisting their leads together.

Lastly wire the switches as shown in the schematic and attach them to the metal plate with a glue of choice, hot glue was chosen because it is easier to remove in case of mistakes. It is recommended to label the switches accordingly. Now wire the LED arrays with each color in parallel and crimp on the connectors, taking note of the correct polarity. Also wire the fans in parallel and crimp on a connector. Lastly after wiring the DC jack to the main switch, glue it to the back panel of the device, other locations are of course possible.

Use thermal glue in combination with hot glue to attach all remaining components to the metal plate, hot glue serves to hold the components while the thermal glue hardens. First attach the heat sink to the center of the plate. Add the shunt resistors as shown in e) and the PCBs to the sides of the heat sink, adding thermal glue to the resistors and linear regulators. Finally organize the wiring, the channels in the heat sink can be used to guide the wires and cable ties and hot glue can be used to hold them in place.

The device can now be set up and tested according to the procedure described below.

## Operation instructions

### Setting up the imager

Set down the imager on a level, solid and vibration free surface. Make sure the imager doesn’t wobble, if necessary insert shims under the corners. Vibrations can cause blurriness during long exposures. Avoid direct sun light to reduce background. The front of the device as well as the fans and heat sinks on the front and back need to be unobstructed or overheating could occur. The same applies to the power supply as it could heat up significantly depending on the settings used. Avoid loose objects that could get sucked into the fans. Plug the power supply into the DC jack at the back of the device while the device is switched off.

Depending on the camera or mobile device used, install the platform using 4M3 screws. Install the required filters by simply pressing the filter into position, a sharp snapping sound should be heard, the filter should be flush with the platform if installed. To remove the filter, press firmly on the two levers on both sides.

### Using the imager

Start by pulling out the tray and putting the object to be imaged into it. If a gel should be imaged, gently lower the gel on one side first and push out any air bubbles trapped under the gel. Then install the optimal filter for the fluorophore used, this should be determined empirically but estimates can be made by comparing the absorption spectrum of the fluorophore to the spectrum of the filters or the dyes tested in this paper, e.g. ethidium bromide has the same spectrum as GelRed and thus similar filters should be tested. Then set the LEDs, determining the ideal setting similarly.

For very bright fluorophores or when used in high concentration, the fluorescence is often well visible with the naked eye. For low concentrations or when the emission wavelength is in the far red, like when using the RG645 filter, it might not be visible without a camera. For documentation, set the camera down on the platform of a custom mount, for bright fluorescence, handheld imaging might be possible with short exposure times. Align the camera so that the whole object is visible. For bright fluorescence, autofocus can be used. Otherwise, set the focus to a predetermined value manually, this value can easily be found by removing any filter and using the bright light to focus on a feature with sharp edges, like the wells in case of a gel. Determine the ideal exposure time empirically, it is recommended to keep the ISO low to avoid noise, if possible, a manual mode should be used so that ISO and exposure can be set by the user. It is recommended to capture multiple images around the ideal exposure value. Avoid disturbing the camera during long exposures. In case that the fans create too many vibrations, they can be temporarily turned off, keep this to a minimum and depending on the cooler, don’t exceed several minutes to avoid overheating, see the thermal testing section.

After use, the sample should be disposed of appropriately and the tray should be cleaned to avoid high background and for safety reasons. Especially with Acridine Orange this step is very important, as the author noticed a strong background in the shape of the gel after it wasn’t properly cleaned off in time. This background was resistant to strong acid and base as well as bleach, so the tray had to be repainted.

## Validation and characterization

### Thermal testing

Thermal testing was carried out using a Fluke 289 multimeter with data logging capabilities with the Fluke 80BK-A thermocouple. The thermocouple was placed at the front side underneath the LED driver PCB in contact with the steel plate and good thermal conductivity was insured using a drop of vacuum pump oil. Recordings were made after letting the device cool down sufficiently until it has equilibrated to room temperature. Recordings were made for a total time of 30 min with data points being captured every second. For further analysis the average temperature of each one second data interval was used, the raw measurements are available in the documentation.

The recording was started at the same time the device was switched on. For the measurements with the fans on, the device was left undisturbed with the fans on for the whole 30 min. For the measurements with the fans off, the device was started with the fans off and the temperature mas monitored. As soon as the temperature reached 70 °C, the fans were turned on and the measurement continued for the remaining time.

**Fig. 8:**
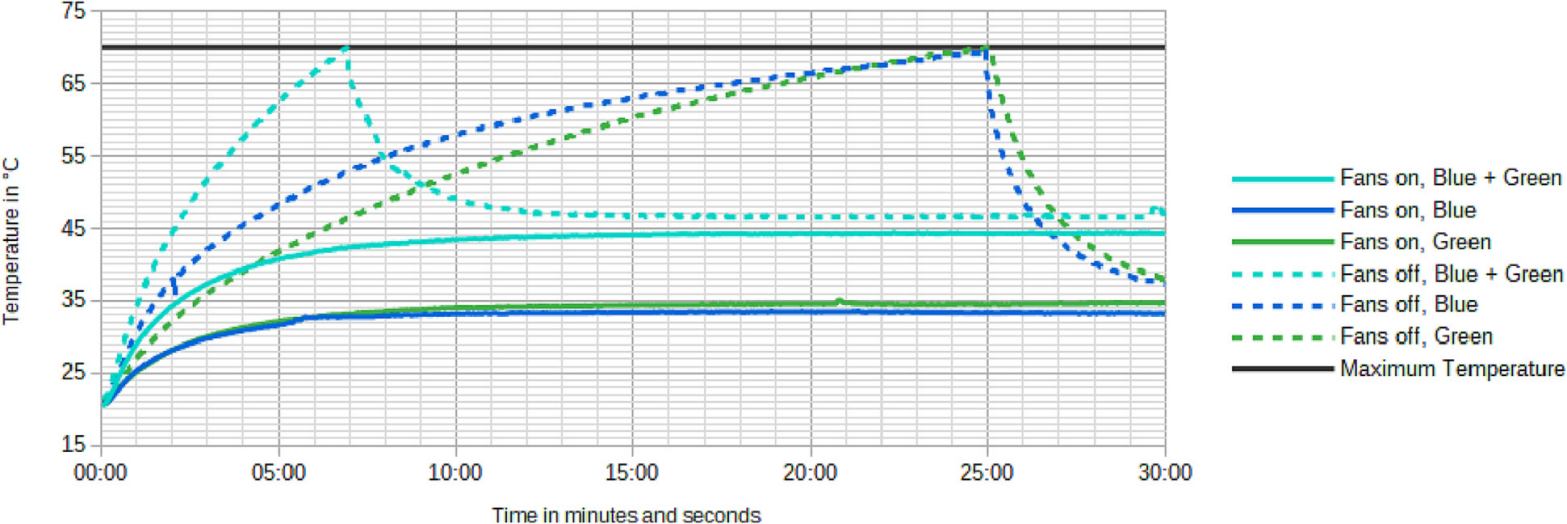
Plot of the temperature versus time, measured as described above with the 70 °C threshold shown as a horizontal line.

This test concluded that the cooling solution is sufficient as the temperature was kept well below 50 °C while the fans were on. It is also safe to turn off the fans for short periods of time, around 5 min for both LED banks and 25 min for only a single set of LEDs, this time can be seen as the time to reach 70 °C in Fig. 8.

### Room light immunity test

When using the device in a laboratory shared with other researchers, it is inconvenient at best, impossible at worst to always turn off the lights when using the imager. Room light immunity is thus an important characteristic. It also separates this device from many inexpensive and simpler builds or using a simple transilluminator. The influence of external light depends on the brightness of the illumination and the background that is inevitable due to excitation light leaking into the camera.

To test this characteristic two images, taken with the room lights on and off, were compared to each other. The tray was loaded into the device empty as this presents the worst case scenario. Images were then taken using the camera of a Samsung Galaxy S10+ with the wide angle camera at exposure times that showed a clearly visible background. This test was carried out with artificial lighting being the only light source as to avoid drift from changes in daylight intensity. Using the app “Physics Toolbox Suite” on the same device, the light intensity was measured at 85 lx with the lights on and 0 lx with the lights off, this should be regarded as an estimation only. Images were then taken using every filter with all applicable LED settings.

The images were then analyzed using ImageJ. The input images were converted to 32bit grayscale and the image with the lights on was divided by the image with the lights off. Using one of the brighter images, the width of the tray was used to calibrate the scale for the images and a 9 × 11 cm ROI, corresponding to the size of the gels used, was saved in the ROI imager. This ROI was then applied to each of the processed images approximately in the center and then the images were cropped and despeckled. The cropped images were exported as a text image. In the text files the spaces were replaced by commas and a whitespace. This allowed them to be imported into a spreadsheet and statistics were carried out and the data was visualized as a boxplot.

These values can be interpreted as the added background in percent of the normal background without any stray light entering the device.

**Fig. 9:**
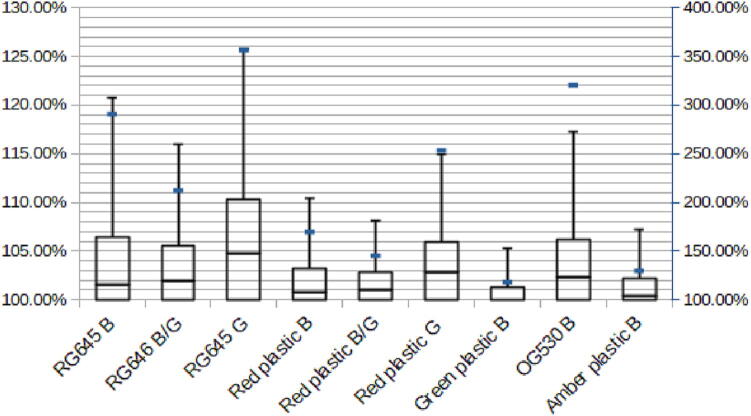
Box plot showing the distribution and intensity of the background caused by the room light. The plot is cut off below 100% as this would mean the background got less by the light and is thus an artifact. It should be noted that the maximum values in blue are referenced to the right, secondary Y-axis. (For interpretation of the references to color in this figure legend, the reader is referred to the web version of this article.)

As can be seen in Fig. 9, even with a filter and LED combination that has very low background and thus would be affected the most by this, the highest median value recorded was still under 5% with much lower values with most filters. However, the maximum value in the worst combination was more than 350%. When looking at the images, a bright spot can be seen, this was caused by light entering the device through a gap and producing a well defined spot that according to this data can be as bright as 3.5 times the usual background.

This represents a worst case scenario with exposure times that are unrealistic during regular use. Apart from direct spots of light on the tray, the device was very resistant to room light. If this presents a problem, black tape can be used to close any gaps at the expense of serviceability. For most test done, this problem was never noticeable as the device was kept in a shelf that shielded it from the direct light. It can be concluded that especially with bright fluorophores and high background from the staining, the device can be used in bright rooms with no noticeable degradation in performance.

### Agarose gel

To test the performance and applicability of the device for its main purpose, two agarose gels were stained with two spectrally very different gel stains. The two gel stains selected were GelRed and Acridine Orange. GelRed is a commonly used gel stain and additionally is spectrally identical to ethidium bromide, which is another common stain. Acridine Orange on it’s own isn’t commonly used for agarose gel electrophoresis, but many commercial gel stains contain it, sometimes without any mention of Acridine Orange [17]. This was also experienced with two other commercial gel stains that were identified as Acridine Orange by their absorption spectrum and using TLC, it can be concluded that Acridine Orange is very widely used and thus representative. Additionally GelGreen has the same absorption spectrum as Acridine Orange.

Agarose gels with a percentage of 1.6% were prepared in 50 ml of 1× LAB buffer [18]. Before pouring the gel stained with GelRed, the stain was added at a concentration of 1x to the hot agarose and mixed. The Acridine Orange stained gel was poured without any gel stain added. The Acridine Orange stained gel was prepared first to avoid cross contamination. In lane 2 to 9 dilutions of Bio-Budget 100 bp + 1.5 kb ladder was loaded. The ladder was diluted by serial dilution with a dilution of 1:1 between lanes, in lane 2, 5 μl of the undiluted ladder was loaded together with Bio-Budget Orange G loading dye. In lanes 3–9 the dilutions were loaded similarly. The gel was run at 300 V corresponding to 20 V/cm in 1× LAB buffer for 15 min. The GelRed stained gel was imaged immediately. The other gel was post-stained with Acridine Orange similarly as previously described [19]. The gel was stained in a 15 µg/ml Acridine Orange solution for 20 min, the longer duration was chosen due to the higher percentage of the gel, it should be noted that dissolving 30 mg of Acridine Orange in 200 ml of water as described results in a 150 µg/ml concentration that lead to unusable staining when tested. The gel was destained in MilliQ water for a total of 3 h, replacing the destaining solution every hour.

Images were captured using a Samsung Galaxy S10+ smartphone with the main camera for each applicable wavelength and using all filters with five exposures. The best image for each filter and stain was selected for further analysis The gel images were cropped to only show the ladders and a montage was made using ImageJ. Documenting the results using a table, the sensitivity of each stain with each filter was graded manually as the faintest band still distinguishable from the background. As grading is subjective, all original and processed images are available in the repository.

**Fig. 10:**
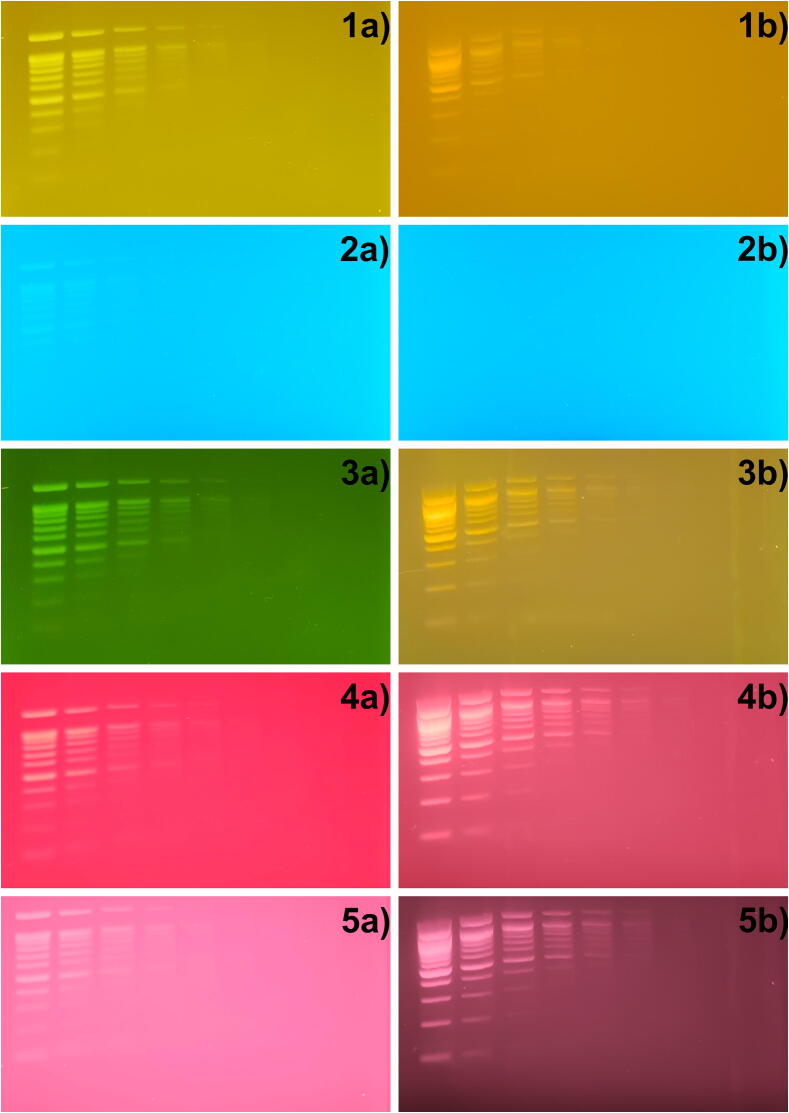
Cropped gel images of the Acridine Orange (Xa)) and GelRed (Xb)) stained gels imaged with different filters. Amber plastic in 1, green plastic in 2, the OG530 filter in 3, red plastic in 4 and the RG645 in 5. (For interpretation of the references to color in this figure legend, the reader is referred to the web version of this article.)

Fig. 10 also gives examples of the performance of the different filters for the two fluorescent stains used.

Additionally the gels were analyzed using ImageJ to plot the intensity of each lane. For this the images were loaded and an FFT filter with a maximum size of 200 pixels and a minimum size of 3 pixels was applied and each lane left to right selected and then plotted. Using these plots the results from the visual test were confirmed and corrected where errors were found, this allowed for higher sensitivity especially with distracting bright background and broadened peaks but care had to be taken not to include false positives from dust, however it should be noted that this showed a higher sensitivity than would be possible with the naked eye. The plots were made available through the repository for verification.

Unsurprisingly the red filters resulted in better sensitivity with GelRed and the filters passing green light worked best with Acridine Orange (As seen in Table 1). With the exception of the green plastic filter however, all filters could be used with all fluorophores, even if the resulting signal was weak and long exposures had to be done. The green plastic filter however, had very high background in both cases, with Acridine Orange the fluorescence was strong enough to still be visible, but at a much lower sensitivity. This can be explained by the overlapping spectrum of the blue plastic filter used on the LEDs. With the right filter the sensitivity was very high with both gel stains, as high as around 3 ng for Acridine Orange and around 1 ng with GelRed. While this is still not as sensitive as the lowest reported value of 0.1 ng [20], one should keep in mind that GelRed should ideally be imaged using UV and performs much worse using visible excitation [1]. Under these circumstances the performance of the device can be regarded as excellent.

**Table 1:** Lowest detectable amount per band divided by gel stain and filter at the optimal LED setting.

Filter used	Sensitivity Acridine Orange	Sensitivity GelRed
Amber plastic	5 ng	8.75 ng
Green plastic	13.75 ng	>200 ng
OG530	3.125 ng	3.125 ng
Red plastic	4.375 ng	1.09375 ng
RG645	4.6875 ng	1.09375 ng

### Uniformity test

A uniform illumination intensity is crucial for accurate quantification and uniform sensitivity. The uniformity was tested both for flat objects like gels and for microtiter plates. To test the uniformity for flat objects, a piece of white paper the size of the tray was laid in the tray and images were captured without a filter with both green and blue LEDs. To test the uniformity in plates, a plate was filled with 200 µl and 400 µl of a dye solution in each well.

The dye solution was chosen to be a 2 µg/ml solution of Eosin Y as it was easily detectable with both green and blue LEDs. The plate was first filled with 200 µl in each well and imaged with blue LEDs with the OG530 filter and green LEDs with the RG645 filter. Then another 200 µl were added and the plate was imaged again. The images were analyzed using ReadPlate3.0 in Fiji and the results saved and imported into a spreadsheet to be analyzed.

To test the uniformity of the white backgrounds, the images were imported into Fiji and using the outline of the tray, the scale was calibrated. Then a 11 × 9 cm ROI was drawn in the center, corresponding to the size of a gel. The image was then cropped according to this ROI and the channels separated. Then the channel corresponding to the LED color was despeckled and a FFT filter with a minimum size of 10 and a maximum size of 100 was applied and the image was exported as a text image. This was opened with a text editor and the empty spaces between values were replaced by comas and white spaces and the file was saved a CSV to be imported into a spreadsheet to be analyzed and visualized together with the data from the plates.

**Fig. 11:**
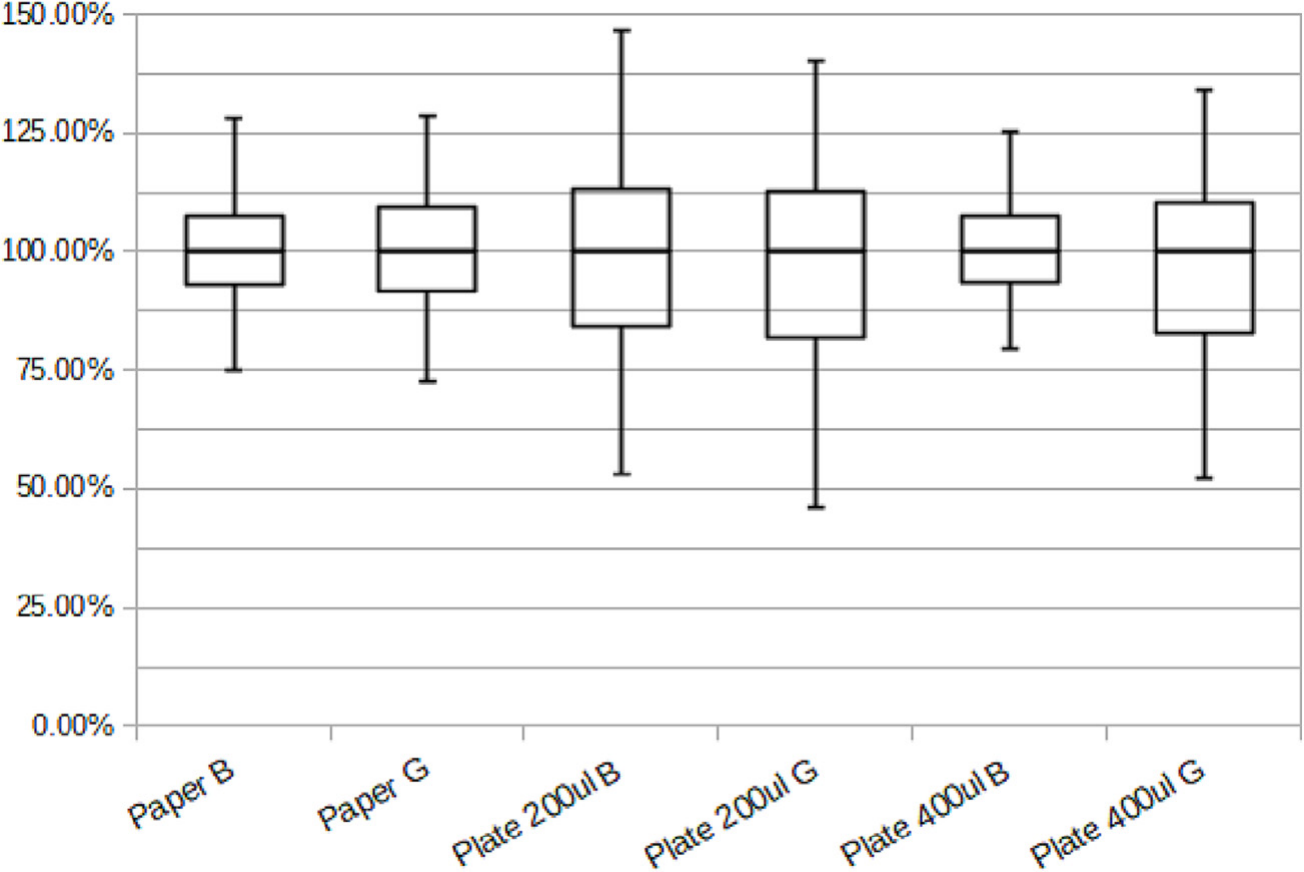
Box plot showing the uniformity of the different objects with different LED settings. The values are normalized to the mean and the whiskers represent the maximum and minimum values.

As can be seen in Fig. 11, for flat objects the uniformity is within +/- 30%. For plates it is very important to fill them with 400 µl of solution as can be seen by the high variability of plates filled with only 200 µl, which is why the plate for the sensitivity test was filled to a volume of 400 µl. This variability should be sufficient for semi-quantitative analysis.

### Sensitivity test

To test the performance of the device and its filters in a more controlled environment without the influence of staining quality, background and scattering by the gel, another test was performed. A selection of 7 fluorophores was diluted in black 96 well plates. The plates were then imaged and the lowest detectable signal was noted.

To start, the fluorophores were diluted with from their respective stock solutions to a concentration of 50 μg/ml at a volume of 1 ml. A 60 %wt glycerin solution was prepared, this concentration was chosen as it neither evaporates or absorbs water from the air [21]. A black 96 well plate (Thermo Scientific Nunc) with a flat bottom was chosen. Transparent plates resulted in high background and should not be used.

First the plate was filled with the glycerin solution, 180 µl in the first column (A1-H1) and 100 µl for the rest of the plate. In the first column (A1–G1) 20 μl of each diluted fluorophore solution as well as a water control in H1 were added and mixed by pipetting. Then serial dilutions with a volume of 100 μl, corresponding to a dilution of 1:1, were performed along the rows, from X1 to X12, for the whole plate. Then 300 μl of the glycerin solution were added to each well and mixed carefully by pipetting, filling the wells to the top. This was done as the excitation light is at a shallow angle and otherwise could not reach the solutions at the bottom of the wells as also seen in the uniformity test.

**Fig. 12:**
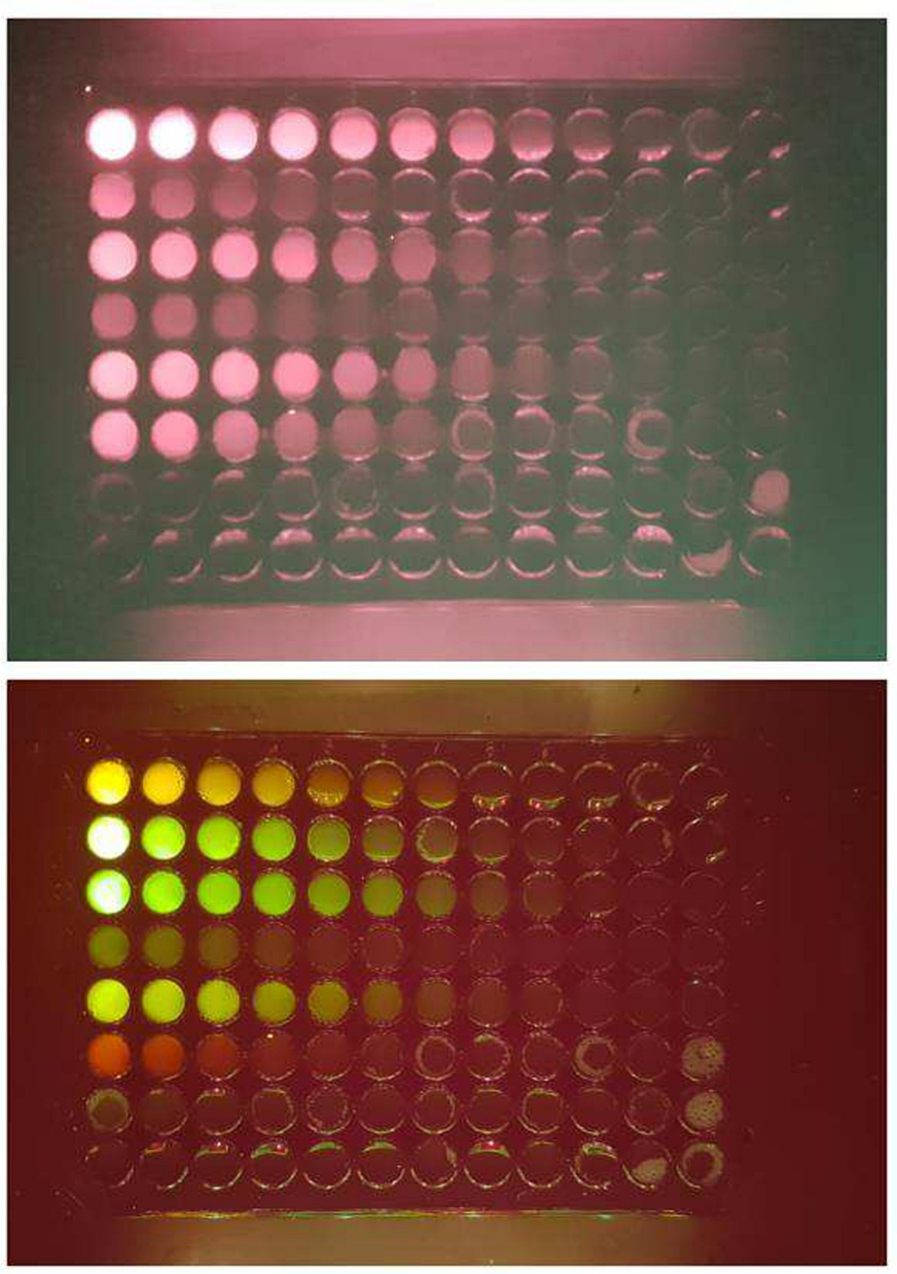
Two examples of the plate imaged, for the top image the RG645 and for the bottom image the OG530 filter was used.

After waiting for some of the bubbles to disappear, the plates were imaged. A Samsung Galaxy S10+ with the main camera and various exposure time were used. For each filter with each applicable LED setting, 5 exposures were made centered around an empirically determined optimal value. Fig. 12 shows two representative images using two different filters showing how different fluorophores have drastically different sensitivities with different filters. The best of the five exposures was selected and the lowest signal distinguishable from the background was recorded in a spreadsheet and visualized.

This showed that apart from the Auramine O, which was very hard to detect and due to its excitation spectrum, every other fluorophore was detectable with almost every filter, with the exception of the green plastic filter, which as already seen with the gels, leaks a lot of excitation light and is unusable for almost all applications. The two fluorophores Rhodamine B and Acridine Orange show that matching the filter and LED setting to the spectrum of the fluorophore is crucial for a high sensitivity. Both fluorophores could be detected at sub-nanogram levels, however, only with the correct filter.

To calculate the intensity of each signal, the Fiji plugin ReadPlate3.0 was used to measure the intensity of the signal. For this the images were loaded into ImageJ and the rotation was adjusted as necessary, then a rectangle from A1 to H12 was drawn and the plugin was run with the settings found in the raw files in the repository. The data was imported into the spreadsheet and the relative intensity was calculated from the raw values by subtracting the blank on H1 from each of the wells in the first column containing the highest concentration of fluorophore according to this formula, where t is the exposure time, and f the aperture, not that a higher aperture corresponds to a lower signal:

$$\mathrm{AU}=1/t*1/ISO*f^{2}*\left(X1-H1\right)$$

Then the results were visualized in a bubble plot with the fluorophores being numbered from 1 to 7 on the X-axis and the sensitivity on a logarithmic Y-axis, the bubble size represents the signal intensity above the background as calculated above. The fluorophore number 1 corresponds to Rhodamine B, 2 corresponds to Acridine Orange, 3 corresponds to Eosin Y, 4 corresponds to Erythrosine B, 5 corresponds to Eosin B, 6 corresponds to Neutral Red and 7 corresponds to Auramine O.

**Fig. 13:**
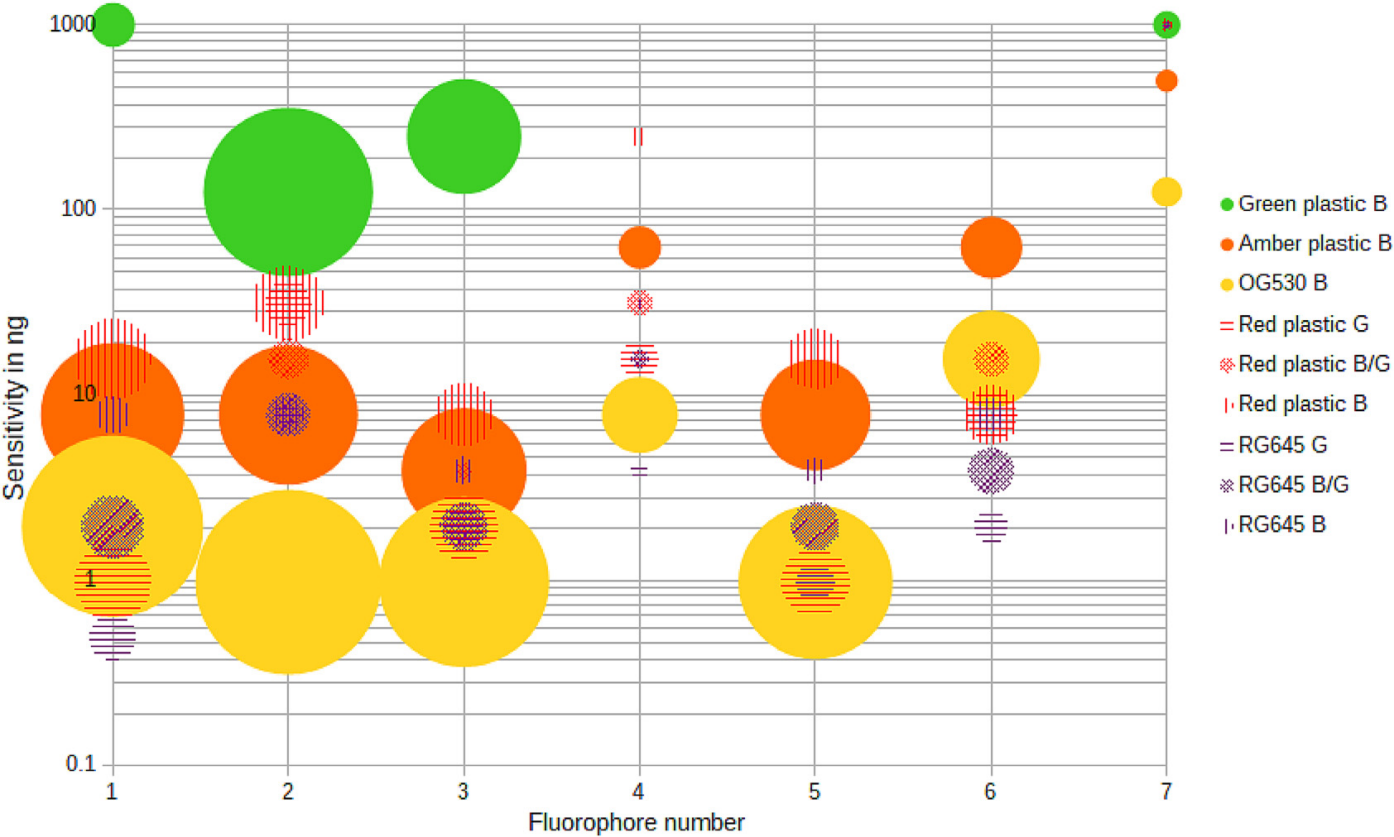
Bubble plot of the different fluorophore and filter combinations with the fluorophores in the X-axis and the sensitivity as a logarithmic Y-axis. The bubble size represents the intensity above the background corrected for exposure with larger bubbles corresponding to higher signals.

Fig. 13 shows the sensitivity values of Table 2 as well as the signal intensity as a bubble plot. The signal intensity dictates the required camera sensitivity and exposure time, with low sensitivities often requiring exposure times of up to 30 s. In cases where two filters have the same sensitivity for a fluorophore, the filter with the strongest signal should be chosen.

**Table 2:** Lowest detectable amount of fluorophore per well divided by filter, LED setting and fluorophores.

Filter and LED setting	Rhodamine B	Acridine Orange	Eosin Y	Erythrosine B	Eosin B	Neutral red	Auramine O
RG645 Blue	7.81 ng	7.81 ng	3.91 ng	31.25 ng	3.91 ng	7.81 ng	–
RG645 Blue/Green	1.95 ng	7.81 ng	1.95 ng	15.63 ng	1.95 ng	3.91 ng	–
RG645 Green	0.49 ng	7.81 ng	1.95 ng	3.91 ng	0.98 ng	1.95 ng	–
Red plastic Blue	15.63 ng	31.25 ng	7.81 ng	250 ng	15.63 ng	7.81 ng	–
Red plastic Blue/Green	1.95 ng	15.63 ng	3.91 ng	31.25 ng	1.95 ng	15.63 ng	–
Red plastic Green	0.98 ng	31.25 ng	1.95 ng	15.63 ng	0.98 ng	7.81 ng	–
OG530 Blue	1.95 ng	0.98 ng	0.98 ng	7.81 ng	0.98 ng	15.63 ng	125 ng
Amber plastic Blue	7.81 ng	7.81 ng	3.91 ng	62.5 ng	7.81 ng	62.5 ng	500 ng
Green plastic Blue	–	125 ng	250 ng	–	–	–	–

This test can be used when evaluating new fluorophores for the device by comparing their absorption spectrum to the ones used in this experiment and helps with selecting the right filter.

### Thin layer chromatography of the dyes

Thin layer chromatography of the dyes used was performed and the plate was imaged using this device. TLC is a powerful tool to estimate the purity of compounds in case of dyes, they can be detected using fluorescence. It can also be used to compare an unknown sample to a known standard, such as comparing a commercial gel stain to a pure sample of Acridine Orange to identify if a gel stain uses that fluorophore. This is a well known method and was also performed by the author to identify two commercial gel stains as Acridine Orange as noted before [17]. Moreover it represents a use case that could not be fulfilled by gel documentation systems that illuminate the sample from below as the TLC plates used here were made on an aluminum backing.

To perform the test, a small, about equal amount of each 50 μg/ml dye solution was spotted on a silica TLC plate (Macherey-Nagel, SIL G) without fluorescence indicator. The plate was then developed in a 5:1 DCM/Methanol solvent mixture in a beaker that was covered to allow for the atmosphere to saturate with solvent vapor. Once the solvent front reached near the top of the plate its position was marked and the plate was allowed to dry before imaging.

**Fig. 14:**
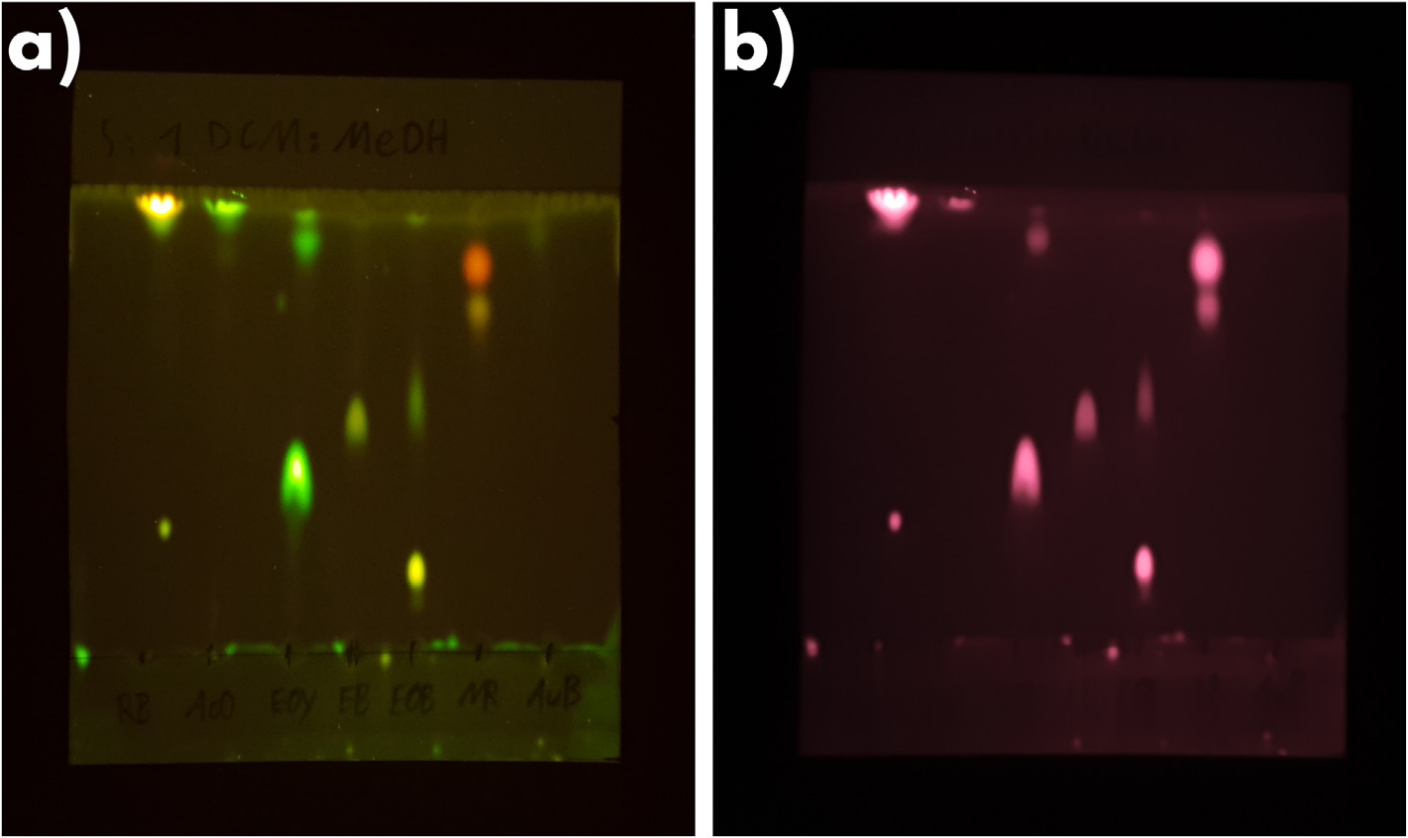
Two examples of the TLC plate imaged with the OG530 filter (a) and the RG645 filter (b). The dyes are abbreviated, from left to right: Rhodamine B, Acridine Orange, Eosin Y, Erythrosine B, Eosin B, Neutral Red and Auramine O. (For interpretation of the references to color in this figure legend, the reader is referred to the web version of this article.)

The plat was imaged using a Samsung Galaxy S10 + smartphone using the main camera at various exposure times and using all filters with all applicable LED settings. The complete data can be found in the repository. Fig. 14 shows two representative images, using two different filters.

While the purity of the dyes used wasn’t important to the tests, this example shows that the device can be used for these types of applications as well that would not be possible using a design that illuminates the sample from the bottom. One such application would be a fluorescent western blot. Similar to a TLC plate, a white membrane is used. The signal is usually much weaker, however, as shown before in the sensitivity test, the device is capable of sub-nanogram sensitivity depending on the fluorophore and filter used.

### Spectra of the filters and tested fluorophores

The spectra of the GelRed gel stain used as well as the commercial glass filters are available online from the manufacturers [22], [23], [24]. However, the colored plastic used as filters were previously uncharacterized. For reference purposes the absorption spectra of the other fluorophores are also included.

The spectra were acquired using a Helios Omega UV–Vis spectrophotometer. The exact parameters are available in the raw files. The filters that were already mounted in their frames were held into the beam path using alligator clips and the unmounted plastic was inserted into the slot of the standard cuvette holder. Spectra of the fluorophores were acquired by diluting them to a final concentration of 10 µg/ml in MilliQ water and measured in a quartz cuvette (Hellma 105.202.008-QS) with a path length of 10 mm. The data was exported as.CSV files and analyzed externally, the raw files are available in the documentation.

## Declaration of Competing Interest

The authors declare that they have no known competing financial interests or personal relationships that could have appeared to influence the work reported in this paper.
